# Dr. J. W. Clowes

**Published:** 1900-11

**Authors:** G. Alden Mills


### DR. J. W. CLOWES.

QUIETLY and almost unknown Dr. J. W. Clowes, one of New York's well-known and much-honored veterans, passed from earth September 9. The doctor had been out of health for the past two years, but kept in sight of his office until about eight months ago, when he sold his residence on Fifth Avenue and removed to a more retired part of the city, One Hundred and Seventeenth Street, facing the attractive neighborhood of the New Columbia College. Although he fitted his office for active service, his strength rapidly failed, so that he was forced to set aside all hopes of again entering into practice.

Dr. Clowes was one that loved his calling in a larger sense than most practitioners. Although he had filled out full fifty years of active practice, he found it hard to say, It is finished, and we may say it was well finished.

Many think seventy-nine years too old for dental practice. Some men grow old mentally more rapidly than others.

Dr. Clowes had two strong features in his practice,—an extreme operation for the removal of the first permanent molars; the large use of an amalgam, "Causeway," for supplying the loss of teeth. This he patented and regarded it of great value. It was a marvel of painstaking industry. His views in regard to methods may be found in dental literature.

In society work he was active, and he had the faculty of expressing himself with clearness.

His office furniture had the air of taste, no expense being spared.

While Dr. Clowes was termed the "millionaire dentist," he always denied the charge, asserting, "I am not, but I have sufficient for the comfortable maintenance of my family."

He studied his profession with his late brother-in-law, J. Smith Dodge, Sr., long known in New York. Dr. Clowes practised in New London, Conn., Columbus, Ga., and in New York. For a year he had an office with the late Dr. Atkinson, in order that he might become familiar with the latter's ideas and methods of practice.

Dr. Clowes's emphatic defence of amalgam has done much to bring it to a better recognition and value as a material for filling and saving teeth. His following has been of a class of people that held very tenaciously to him so long as he was able to serve them. He was fond of exhibiting his many letters received from grateful patients. Who can say that these do not bring much pleasure and solid comfort to a faithful practitioner?

All who knew Dr. Clowes can speak truthfully of his sincerity and faithfulness, while they may not altogether endorse some of his extreme views in practice.

The death of Dr. Clowes was not generally known owing to the general absence from the city at this period of the year.

It is but little that we can say of such a man as we follow him to the tomb. He has had his meed of praise in many ways. Some are apt to think, later in life, that they are not fully appreciated, but all such worthy workers as Dr. Clowes have a full reward in the consciousness of having done their full duty.

G. ALDEN MILLS.

